# (*E*)-4-Nitro­benzaldehyde oxime

**DOI:** 10.1107/S1600536810013978

**Published:** 2010-04-21

**Authors:** Asghar Abbas, Safdar Hussain, Noureen Hafeez, Amir Badshah, Aurangzeb Hasan, Kong Mun Lo

**Affiliations:** a Department of Chemistry, Quaid-i-Azam University, Islamabad 45320, Pakistan; b Department of Forensic Medicine & Toxicology, National University of Sciences & Technology, Islamabad, Pakistan; c Department of Chemistry, University of Malaya, 50603 Kuala Lumpur, Malaysia

## Abstract

In the title compound, C_7_H_6_N_2_O_3_, the planes containing the CNO and ONO atoms subtend dihedral angles of 5.47 (5) and 8.31 (5)°, respectively, with the benzene ring. In the crystal structure, inter­molecular O—H⋯N hydrogen bonds link the mol­ecules into centrosymmetric dimers with an *R*_2_^2^(6) graph-set motif.

## Related literature

For oximes as therapeutic agents in organophospho­rus poisoning, see: Jokanovic *et al.* (2009 ▶); Marrs *et al.* (2006 ▶). For their use as protecting groups in organic synthesis, see: Greene *et al.* (1999 ▶); Shinada *et al.* (1995 ▶). For graph-set notation, see: Etter *et al.* (1990 ▶); Bernstein *et al.* (1995 ▶). For bond lengths in similar structures, see: Xing, Ding *et al.* (2007 ▶); Xing, Wang *et al.* (2007 ▶). 
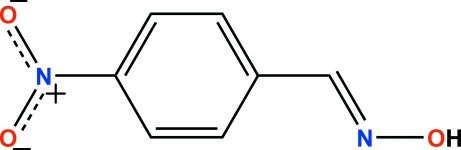


## Experimental

### 

#### Crystal data

C_7_H_6_N_2_O_3_
*M* *_r_* = 166.14
Monoclinic, 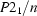

*a* = 3.7737 (2) Å
*b* = 7.0363 (3) Å
*c* = 28.6651 (14) Å
β = 91.237 (3)°
*V* = 760.96 (6) Å^3^
*Z* = 4
Mo *K*α radiation
μ = 0.12 mm^−1^
*T* = 296 K
0.49 × 0.41 × 0.16 mm

#### Data collection

Bruker APEXII CCD area-detector diffractometer
Absorption correction: multi-scan (*SADABS*; Sheldrick, 1996 ▶) *T*_min_ = 0.945, *T*_max_ = 0.982
7222 measured reflections
1869 independent reflections
1340 reflections with *I* > 2σ(*I*)
*R*_int_ = 0.031

#### Refinement

*R*[*F* ^2^ > 2σ(*F* ^2^)] = 0.066
*wR*(*F* ^2^) = 0.175
*S* = 1.09
1869 reflections
110 parameters
H-atom parameters constrained
Δρ_max_ = 0.20 e Å^−3^
Δρ_min_ = −0.20 e Å^−3^

### 

Data collection: *APEX2* (Bruker, 2008 ▶); cell refinement: *SAINT* (Bruker, 2008 ▶); data reduction: *SAINT*; program(s) used to solve structure: *SHELXS97* (Sheldrick, 2008 ▶); program(s) used to refine structure: *SHELXL97* (Sheldrick, 2008 ▶); molecular graphics: *X-SEED* (Barbour, 2001 ▶); software used to prepare material for publication: *publCIF* (Westrip, 2010 ▶).

## Supplementary Material

Crystal structure: contains datablocks I, global. DOI: 10.1107/S1600536810013978/hg2672sup1.cif

Structure factors: contains datablocks I. DOI: 10.1107/S1600536810013978/hg2672Isup2.hkl

Additional supplementary materials:  crystallographic information; 3D view; checkCIF report

## Figures and Tables

**Table 1:** Hydrogen-bond geometry (Å, °)

*D*—H⋯*A*	*D*—H	H⋯*A*	*D*⋯*A*	*D*—H⋯*A*
O3—H3⋯N2^i^	0.82	2.12	2.841 (3)	146

Symmetry code: (i) 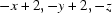.

**Fig. 1.**
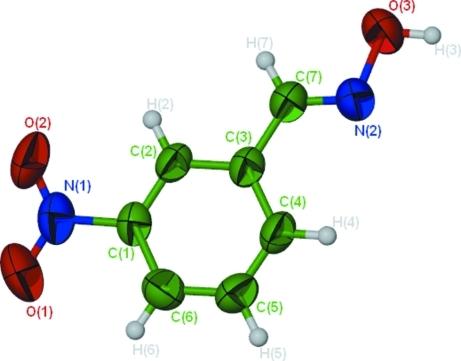
Molecular structure of (I) showing atom numbering scheme. Displacement ellipsoids are drawn at the 50% probability level.

**Fig. 2.**
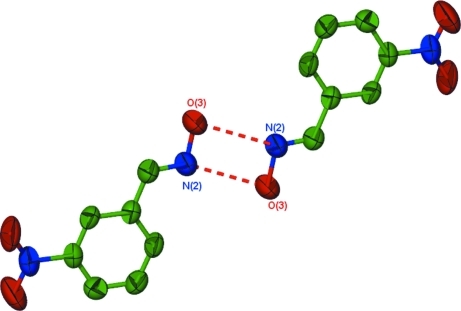
Hydrogen bonds shown as dashed lines, forming dimers through R22(6) graph set motif.

## supplementary crystallographic information

### Comment

Thousands of deaths are caused by acute organophosphorus pesticide poisoning each year. Oximes are accepted therapeutic agents in organophosphorus poisoning (Jokanovic *et al.*, 2009, Marrs *et al.*, 2006). Oximes can act as useful protecting groups (Greene *et al.*, 1999) and have served for the protection of carbonyl groups in the syntheses of erythromycin derivatives and perhydrohistrionicotoxin (Shinada *et al.*, 1995). Oximes are also used for the purification and characterization of carbonyl compounds. As part of our interest in the study of oxime derivatives, we report here the crystal structure of the title compound (I). A depiction of the molecule is given in Fig. 1. In the crystal structure of the title compound, molecules are connected via intermolecular O—H···N hydrogen bonds (see Table 1 and Fig. 2) to form two-dimensional dimers. The oxime group has an *E* configuration [C3—C7—N2—O3 = 179.1 (2)°] and the planes containing the CNO and ONO atoms subtend dihedral angles of 5.47 (5)° and 8.31 (5)° with the phenyl C(1–6) ring. which is less than that reported for similar structures (Xing & Ding *et al.*, 2007; Xing & Wang *et al.*, 2007). Each molecule is connected to a symmetry-related molecule through an inversion center by O—H···N hydrogen bonds, building an R_2_^2^(6) graph-set motif in Fig. 2 (Etter *et al.*, 1990; Bernstein *et al.*, 1995; ).

### Experimental

To a warm solution of 4-nitrobenzaldehyde (0.907 g , 0.005 mol) in 25 ml e thanol, hydroxylamine hydrochloride (0.417 g, 0.006 mol) and sodium acetate trihydrate (2.04 g, 0.015 mol) were added and the mixture was heated under reflux until completion of the reaction. The concentrated reaction mixture was cooled down and water was added. The precipitated oxime was separated by filtration, washed with excess of water and dried. The crude product was recrystallized from ethanol to get the title compound (I).

### Refinement

All H atoms were placed in calculated position and treated as riding on their parent atoms with C—H = 0.93Å or O—H = 0.82Å with U_iso_(H) = 1.2U_eq_(C) or 1.5U_eq_(O) for the hydroxyl H atom.

### Crystal data

**Table:**

C_7_H_6_N_2_O_3_	*F*(000) = 344
*M**_r_* = 166.14	*D*_x_ = 1.450 Mg m^−^^3^
Monoclinic, *P*2_1_/*n*	Mo *K*α radiation, λ = 0.71073 Å
Hall symbol: -P 2yn	Cell parameters from 1727 reflections
*a* = 3.7737 (2) Å	θ = 2.8–25.1°
*b* = 7.0363 (3) Å	µ = 0.12 mm^−^^1^
*c* = 28.6651 (14) Å	*T* = 296 K
β = 91.237 (3)°	Block, colorless
*V* = 760.96 (6) Å^3^	0.49 × 0.41 × 0.16 mm
*Z* = 4	

### Data collection

**Table:**

Bruker APEXII CCD area-detector diffractometer	1869 independent reflections
Radiation source: fine-focus sealed tube	1340 reflections with *I* > 2σ(*I*)
graphite	*R*_int_ = 0.031
ω scans	θ_max_ = 28.2°, θ_min_ = 2.8°
Absorption correction: multi-scan (*SADABS*; Sheldrick, 1996)	*h* = −5→4
*T*_min_ = 0.945, *T*_max_ = 0.982	*k* = −9→9
7222 measured reflections	*l* = −38→37

### Refinement

**Table:**

Refinement on *F*^2^	Primary atom site location: structure-invariant direct methods
Least-squares matrix: full	Secondary atom site location: difference Fourier map
*R*[*F*^2^ > 2σ(*F*^2^)] = 0.066	Hydrogen site location: inferred from neighbouring sites
*wR*(*F*^2^) = 0.175	H-atom parameters constrained
*S* = 1.09	*w* = 1/[σ^2^(*F*_o_^2^) + (0.060*P*)^2^ + 0.435*P*] where *P* = (*F*_o_^2^ + 2*F*_c_^2^)/3
1869 reflections	(Δ/σ)_max_ < 0.001
110 parameters	Δρ_max_ = 0.20 e Å^−^^3^
0 restraints	Δρ_min_ = −0.20 e Å^−^^3^

### Special details

**Table:**

Geometry. All esds (except the esd in the dihedral angle between two l.s. planes) are estimated using the full covariance matrix. The cell esds are taken into account individually in the estimation of esds in distances, angles and torsion angles; correlations between esds in cell parameters are only used when they are defined by crystal symmetry. An approximate (isotropic) treatment of cell esds is used for estimating esds involving l.s. planes.
Refinement. Refinement of *F*^2^ against ALL reflections. The weighted *R*-factor wR and goodness of fit *S* are based on *F*^2^, conventional *R*-factors *R* are based on *F*, with *F* set to zero for negative *F*^2^. The threshold expression of *F*^2^ > σ(*F*^2^) is used only for calculating *R*-factors(gt) etc. and is not relevant to the choice of reflections for refinement. *R*-factors based on *F*^2^ are statistically about twice as large as those based on *F*, and *R*- factors based on ALL data will be even larger.

### Fractional atomic coordinates and isotropic or equivalent isotropic displacement parameters (Å^2^)

**Table:**

	*x*	*y*	*z*	*U*_iso_*/*U*_eq_	
O3	0.9103 (8)	0.7939 (3)	−0.02398 (6)	0.0848 (8)	
H3	0.9673	0.8950	−0.0359	0.127*	
O2	0.2494 (7)	0.1865 (3)	0.14956 (8)	0.0857 (8)	
N1	0.3651 (6)	0.3009 (3)	0.17705 (8)	0.0575 (6)	
N2	0.8746 (6)	0.8191 (3)	0.02418 (7)	0.0562 (6)	
C1	0.5229 (6)	0.4774 (3)	0.15885 (8)	0.0425 (5)	
C2	0.5707 (6)	0.4913 (3)	0.11163 (8)	0.0426 (5)	
H2	0.5092	0.3912	0.0919	0.051*	
C3	0.7126 (6)	0.6576 (3)	0.09400 (8)	0.0415 (5)	
C7	0.7669 (7)	0.6696 (3)	0.04381 (9)	0.0529 (6)	
H7	0.7205	0.5629	0.0255	0.063*	
C6	0.6116 (7)	0.6200 (3)	0.18954 (8)	0.0509 (6)	
H6	0.5788	0.6059	0.2214	0.061*	
C5	0.7516 (7)	0.7856 (3)	0.17149 (9)	0.0549 (6)	
H5	0.8135	0.8850	0.1914	0.066*	
C4	0.7997 (6)	0.8040 (3)	0.12444 (8)	0.0474 (6)	
H4	0.8922	0.9165	0.1128	0.057*	
O1	0.3545 (8)	0.2798 (4)	0.21888 (8)	0.1044 (9)	

### Atomic displacement parameters (Å^2^)

**Table:**

	*U*^11^	*U*^22^	*U*^33^	*U*^12^	*U*^13^	*U*^23^
O3	0.143 (2)	0.0654 (13)	0.0469 (11)	−0.0300 (13)	0.0171 (12)	0.0024 (9)
O2	0.1130 (19)	0.0472 (11)	0.0966 (17)	−0.0354 (12)	−0.0037 (13)	0.0100 (10)
N1	0.0583 (13)	0.0447 (11)	0.0694 (15)	−0.0043 (10)	0.0033 (11)	0.0160 (10)
N2	0.0732 (15)	0.0490 (11)	0.0467 (11)	−0.0115 (10)	0.0070 (10)	0.0029 (9)
C1	0.0405 (11)	0.0353 (10)	0.0518 (13)	−0.0014 (9)	0.0023 (9)	0.0071 (9)
C2	0.0467 (12)	0.0318 (10)	0.0491 (12)	−0.0054 (9)	−0.0020 (9)	−0.0028 (9)
C3	0.0432 (12)	0.0335 (10)	0.0478 (12)	−0.0031 (9)	−0.0004 (9)	0.0015 (9)
C7	0.0677 (16)	0.0416 (12)	0.0496 (14)	−0.0128 (11)	0.0028 (11)	−0.0025 (10)
C6	0.0586 (15)	0.0501 (13)	0.0442 (12)	0.0019 (11)	0.0022 (10)	0.0012 (10)
C5	0.0706 (17)	0.0410 (12)	0.0528 (14)	−0.0082 (11)	−0.0034 (12)	−0.0077 (10)
C4	0.0543 (14)	0.0332 (10)	0.0544 (14)	−0.0095 (9)	−0.0020 (10)	0.0008 (9)
O1	0.155 (3)	0.0918 (17)	0.0674 (15)	−0.0365 (17)	0.0135 (14)	0.0309 (12)

### Geometric parameters (Å, °)

**Table:**

O3—N2	1.401 (3)	C2—H2	0.9300
O3—H3	0.8200	C3—C4	1.385 (3)
O2—N1	1.202 (3)	C3—C7	1.460 (3)
N1—O1	1.210 (3)	C7—H7	0.9300
N1—C1	1.477 (3)	C6—C5	1.385 (3)
N2—C7	1.264 (3)	C6—H6	0.9300
C1—C6	1.371 (3)	C5—C4	1.371 (3)
C1—C2	1.373 (3)	C5—H5	0.9300
C2—C3	1.387 (3)	C4—H4	0.9300
			
N2—O3—H3	109.5	C2—C3—C7	118.11 (19)
O2—N1—O1	123.2 (2)	N2—C7—C3	122.7 (2)
O2—N1—C1	118.4 (2)	N2—C7—H7	118.7
O1—N1—C1	118.4 (2)	C3—C7—H7	118.7
C7—N2—O3	111.8 (2)	C1—C6—C5	117.8 (2)
C6—C1—C2	123.0 (2)	C1—C6—H6	121.1
C6—C1—N1	118.9 (2)	C5—C6—H6	121.1
C2—C1—N1	118.1 (2)	C4—C5—C6	120.4 (2)
C1—C2—C3	118.63 (19)	C4—C5—H5	119.8
C1—C2—H2	120.7	C6—C5—H5	119.8
C3—C2—H2	120.7	C5—C4—C3	121.0 (2)
C4—C3—C2	119.1 (2)	C5—C4—H4	119.5
C4—C3—C7	122.78 (19)	C3—C4—H4	119.5
			
O2—N1—C1—C6	171.2 (2)	C4—C3—C7—N2	−5.0 (4)
O1—N1—C1—C6	−7.8 (4)	C2—C3—C7—N2	175.6 (3)
O2—N1—C1—C2	−8.2 (3)	C2—C1—C6—C5	0.8 (4)
O1—N1—C1—C2	172.8 (3)	N1—C1—C6—C5	−178.6 (2)
C6—C1—C2—C3	−0.5 (3)	C1—C6—C5—C4	−0.2 (4)
N1—C1—C2—C3	178.8 (2)	C6—C5—C4—C3	−0.5 (4)
C1—C2—C3—C4	−0.3 (3)	C2—C3—C4—C5	0.8 (4)
C1—C2—C3—C7	179.1 (2)	C7—C3—C4—C5	−178.6 (2)
O3—N2—C7—C3	179.1 (2)		

### Hydrogen-bond geometry (Å, °)

**Table:**

*D*—H···*A*	*D*—H	H···*A*	*D*···*A*	*D*—H···*A*
O3—H3···N2^i^	0.82	2.12	2.841 (3)	146

Symmetry codes: (i) −*x*+2, −*y*+2, −*z*.
